# Supplementary figures and images for: Design of a robust active fuzzy parallel distributed compensation anti-vibration controller for a hand-glove system
Source: PeerJ Comput Sci. 2021 Oct 29;7:e756. doi: 10.7717/peerj-cs.756 (PMC8576561; doi:10.7717/peerj-cs.756)

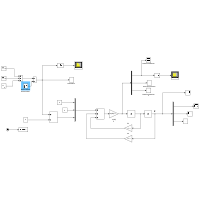

Supplement: Supplemental Information 2 — The Simulation of the passive model. the data needed for this simulation are saved in the system parameters mfile. [file peerj-cs-07-756-s002.slx › metadata/thumbnail.png]

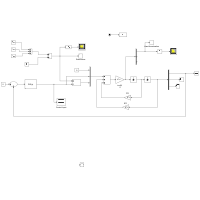

Supplement: Supplemental Information 3 — The Simulink file for the simulation of the glove-hand system model with PID controller. It's outcome is the vibration received by user. [file peerj-cs-07-756-s003.slx › metadata/thumbnail.png]

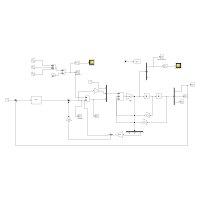

Supplement: Supplemental Information 5 — The file shows the Simulink of the glove-hand system and the designed active force controller. [file peerj-cs-07-756-s005.slx › metadata/thumbnail.png]

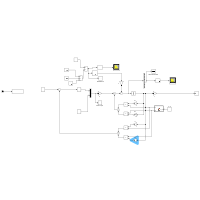

Supplement: Supplemental Information 8 — The simulink representation of the T-S model of the glove-hand system and the PDC controller are given in this file. The result represents the vibration at the user's hand. [file peerj-cs-07-756-s008.slx › metadata/thumbnail.png]
